# Characteristics of Adolescents with and without a Family History of Substance Use Disorder from a Minority Cohort

**DOI:** 10.3390/children11060671

**Published:** 2024-05-31

**Authors:** Keely Cheslack-Postava, Yael M. Cycowicz, Diana V. Rodriguez-Moreno, Lawrence V. Amsel, George J. Musa, Megan Ryan, Michaeline Bresnahan, Huilan Tang, Lupo Geronazzo-Alman, Adam Bisaga, Zhishun Wang, Xiaofu He, Christina W. Hoven

**Affiliations:** 1 New York State Psychiatric Institute, New York, NY 10032, USA; 2 Department of Psychiatry, Vagelos College of Physicians and Surgeons, Columbia University, New York, NY 10032, USA; 3 Department of Epidemiology, Mailman School of Public Health, Columbia University, New York, NY 10032, USA

**Keywords:** fmily history, substance use disorder, minority cohort, family environment

## Abstract

Family history (FH+) of substance use disorder (SUD) is an established risk factor for offspring SUD. The extent to which offspring psychological traits or the family environment, each of which may be relevant to familial transmission of SUD risk, vary by FH+ in socioeconomically disadvantaged populations is less clear. We compared the family/social environmental and psychological characteristics of 73 FH+ and 69 FH- youth ages 12–16, from a study of parental criminal justice system involvement in a primarily low-income, minority urban population. A latent profile analysis (LPA) empirically identified groups of subjects with similar psychological characteristics, which were then compared by FH+. FH+ youths were found to have greater mean household size, greater parental psychological aggression, and a higher mean number of adverse childhood experiences, even without considering parental SUD. FH+ individuals had lower report card grades according to parental report and were more likely to have a history of externalizing disorders than FH- individuals. However, FH+ was not significantly associated with many psychological characteristics or with the class membership from the LPA. In conclusion, among a population of low-income, minority urban youth, FH+ was associated with differences in the family environment and only subtle differences in individual psychological characteristics.

## 1. Introduction

One of the well-established risk factors for substance use disorder (SUD) is a family history (FH+) of SUD [1,2,3]. Familial transmission of SUD likely occurs through a complex interaction of genetic, behavioral, and environmental mechanisms [4,5,6,7]. While the genetic risk for SUD is by itself high, lower socioeconomic status (SES) [8] and environmental factors increase vulnerability to SUD, including via epigenetic modifications [9]. Importantly, childhood family environment can play a key role in increasing SUD risk through exposure to chronic or severe early life stressors. These include maltreatment [10,11], exposure to a dysfunctional family system, or to problematic parenting relationships [3,12,13]. Furthermore, familial transmission of behavioral disinhibition, reduced cognitive control, heightened reward sensitivity, impulsivity, risk-seeking, emotional dysregulation, and antisocial or externalizing behaviors [14] also contribute to SUD risk [15,16,17]. 

While much of the literature has focused on the FH+ of adults with SUD, interest in understanding the impact of FH+ on adolescents prior to their substance use initiation has increased. For example, Tarter and colleagues reported a higher disinhibition behavior index among 10–12 year old boys with FH+ compared to age matched FH- boys [18]. They concluded that the disinhibition behavior index, derived from measures of affect, behavior and cognition, is a contributing factor to the greater risk that FH+ boys have for an early age of substance use onset and subsequent progression to SUD. A larger research study of 10–12 year old boys and girls reported more emotional and behavioral symptoms, worse family relationships, and more deviant peer relationships among the FH+ children [19]. While both of these studies demonstrated that maladaptive behavior at a young age, and prior to substance use initiation, is associated with greater risk for SUD, they had similar methodological limitations. The FH+ and FH- groups differed by demographic characteristics such as race/ethnicity, and parental marital and socioeconomic status, as well as by IQ and educational attainment. These characteristics could account for the differences found rather than their actual FH+/ FH status. 

To overcome these methodological issues, we are conducting the Adolescent Imaging Study (AIS) to longitudinally examine the relationship of FH+ on substance use initiation and trajectories during the adolescent years, as well as to investigate the development of cognitive control and reward sensitivity and their related brain circuitry in relationship to FH+ status [20]. Our sample is based on a low-income, largely minority cohort from an urban community disproportionately affected by the criminal justice system, so the overall social and neighborhood environment are similar across all participants [21]. Here, we: (a) describe the AIS participants, comparing their demographic characteristics and family/social environments by FH+; and (b) determine whether there are baseline differences between FH+ and FH- participants in self-reported psychological and functional characteristics after accounting for demographics and the family environment. 

We examined a number of related behaviors and characteristics that have been suggested as conferring higher risk for SUD, including the prevalence of externalizing disorders, [14,22] antisocial behaviors, internalizing traits including anxiety [8], and higher levels of neuroticism [23]. Moreover, there have been reports, among FH+ offspring, of inattention [24], higher self- and parent-reported impulsivity [22,24], reward/punishment sensitivity [25], negative emotionality [26], lower academic achievement [23], and lower IQ scores [8]. Thus, we chose participants with quite similar demographic and environmental characteristics but differing in their FH+, and examined whether psychosocial and personal characteristics still differed across FH+/FH- groups. 

Because individuals with SUD also exhibit the behaviors described above, studies of adult offspring of FH+ families have been unable to differentiate if these behaviors constitute a consequence of SUD or a predisposition for SUD. By studying adolescents’ behavior prior to significant SU initiation, we are able to compare across FH+/FH- groups and study these behaviors before SU initiation. 

## 2. Materials and Methods

### 2.1. Participants and Procedures

The study population was recruited as a subsample of two epidemiological studies, the Stress & Justice (S&J) studies, that examined the effect on children of their parents’ criminal justice system involvement (CJSI). The S&J studies’ population lives predominantly within low-socioeconomic, high crime areas of the South Bronx, with the Bronx being one of the five boroughs that make up New York City. The population of the South Bronx is comprised predominantly of minoritized racial and ethnic groups, with approximately four fifths self-identified as Hispanic or as African-American, not Hispanic. The South Bronx includes one of the highest poverty districts in the United States and has high levels of rent burden, housing defects, and air pollution. The S&J studies included index families in which the participating parent or guardian had experienced a recent arrest (CJSI+), and control families where the parent or guardian was not arrested within the lifetime of the child/children (though they may have been arrested prior to the child’s birth). There were 418 index families, comprising a parent/guardian and 1–2 of their children aged 9–15, at the time of enrollment. The 344 control families were selected as being in close geographic proximity to the index families, and were gender matched to the index parents, while the control children were frequency matched by age and gender to the index child. The S&J Studies collected data from parent and child participants during 2 waves, approximately 18 months apart. 

The AIS study (constituting wave 3 of the S&J studies) began enrollment approximately 13 months after the conclusion of wave 2. The AIS study included an interview and MRI component, in a subsample of 242 children living within a 100-mile radius of New York City between the ages of 12–14 (later revised to 12–16) at the time of enrollment in wave 3, who were recruited via a letter of invitation. The AIS study was designed to include 50% of the child sample as having a parent with probable lifetime SUD, based on the Composite International Diagnostic Interview (CIDI) [27] assessments collected in the S&J Study (see below). Recruiters followed up with screening interviews to determine the child’s eligibility to participate in the MRI portion of the study and as not having a SUD diagnosis. The current report includes 142 families who completed the baseline interview and had sufficient information for classification as FH+ or FH- (see below). Parents received $70 in cash for completion of the interviews and children received a $50 gift card. The levels were set so as not to constitute an undue influence on the decision to participate. The study received approval from the Institutional Review Board of the New York State Psychiatric Institute.

### 2.2. Measures

All measures were derived from the current wave 3 AIS study except where otherwise noted.

Family history of substance use. Participant families were classified as family history positive (FH+) for substance use disorder if: (a) the parent/guardian participant in the S&J Study (waves 1 & 2), was classified with probable lifetime DSM-IV alcohol or drug abuse or dependence based on their responses to the Composite International Diagnostic Interview (CIDI) [27]; or (b) if either the primary parent/guardian (referred to below as “parent”) or other adult participant in the current study was classified with probable lifetime alcohol or drug abuse or dependence based on the CIDI administered at the eligibility screening (wave 3). 

Demographics. The youth’s age, gender, and race/ethnicity were self-reported. The participant parent reported on their own level of education and current employment status, and on the annual household income of the child’s family. 

Youth Family and Social Environment. 

Composition of the household (numbers of people, total and under age 18, currently living in the household; and family membership status, including the biological mother and father) was reported by the youth.

Parental monitoring. Parent monitoring behaviors (how frequently the parent knows where her/his child is, tells the child when she/he should be home, etc.) and closeness of parent-child interaction (how proud the parent is of her/his child, how much they trust their child, etc.) were captured using a scale adapted from Patterson [28]. The parent rated each statement using a scale ranging from “Never/Almost Never” to “Very Often.” 

Parent-Child Conflict Tactics Scale-Short Form (CTS-PC-SF) [29,30]. This scale measures parent communication style with the child (i.e., when the child makes a mistake, are they yelled/screamed at? Does the parent explain why what they did was wrong?), discipline behaviors (e.g., how frequently child is put in time out/sent to room) and corporeal punishment of child (e.g., hitting child). The parent rates how frequently they engage in each behavior using a scale ranging from “More than 20 times in the Past Year” to “Never”.

Perceived availability of substances. Youth responded to the question “How difficult do you think it would be for you to get each of the following types of drugs, if you wanted some?” for a list of specific drugs. Responses ranged from “probably impossible” to “very easy”. As in prior work, responses were dichotomized with “fairly easy” or “very easy” considered as a positive response [31].

Perceived harm of substances. For specific scenarios of substance use, youth were asked the extent to which people risked harming themselves physically and in other ways. Responses ranged from “no risk” to “great risk”. Consistent with previous reports [31], responses were dichotomized as great risk versus less than great risk.

Peer relations. Child report of relations with peers in terms of: (a) feelings of belonging, (b) feelings of being liked, and (c) feelings of getting along with peers, as adapted for the Boricua Youth Study [32]. Items were rated on a scale ranging from “Rarely or Never” to “Often or all of time.”

Adverse Childhood Experiences (ACEs) [33] were assessed as a cumulative count of 10 categories of negative experiences based on child and parent report at either of the first two S&J study waves. These included verbal, physical, or sexual abuse; neglect; parental divorce or separation; domestic violence; parental SUD, mental illness, or incarceration; and bullying. A count of ACEs excluding parental SUD was also examined, given that it varied by design between groups.

Youth psychological characteristics and functioning. The youth reported their current grade level and grades on their last report card; separately, parents reported the child’s grades during the past year. 

Child’s history of psychopathology was assessed based on the Diagnostic Interview Schedule for Children Version IV (DISC-IV) [34] administered at S&J waves 1 and 2, with probable diagnoses based on both parent and child report of the child’s symptoms. Children were classified as positive for history of an internalizing disorder based on a probable diagnosis, at either wave, of either: panic disorder, separation anxiety, generalized anxiety disorder, major depressive disorder, post-traumatic stress disorder, or agoraphobia. They were classified as positive for a history of an externalizing disorder based on probable diagnosis of oppositional defiant disorder or conduct disorder.

Child’s history of substance use was considered positive if they indicated: ever having had at least one drink of alcohol (more than a few sips), ever having smoked a whole cigarette, ever having used marijuana, or ever having used any of the following: cocaine, inhalants, heroin, methamphetamines, ecstasy, non-prescribed steroids, misuse of prescription drugs, or injection of illegal drugs. A history of regular substance use was based on a report, at wave 3, of ever having: consumed ≥ 6 drinks of alcohol, or used marijuana ≥6 times, or used a combination of ≥3 other substances (excluding cigarettes). 

K-BIT IQ Composite Standard Score (KBIT-2) [35] is a standardized composite measure of raw verbal (crystallized) and non-verbal (fluid) intelligence, with a mean of 100 and a standard deviation of 15. It was administered to child participants during the S&J study wave 2. 

State-Trait Anxiety Inventory (STAI) [36] is comprised of two scales measuring state anxiety (at the time of measurement) and trait anxiety (usual feelings of anxiety) in participants. High state and trait anxiety scores have previously been associated with adolescent substance use [37]. 

NICHQ Vanderbilt Assessment Scale of ADHD (Vanderbilt) is a parental report of a child’s ADHD symptoms, items are from “never” to “very often.” It screens for Predominantly Inattentive and Predominantly Hyperactive/Impulsive subtypes [38]. 

The Behavioral Inhibition System (BIS) and the Behavioral Activation System (BAS) [39] scales were administered to the child and measure motivation towards goal-oriented outcomes (BAS) and away from negative outcomes (BIS). The BAS consists of three sub-scales: drive (motivation towards goal-oriented behavior), reward responsiveness (salience of rewards) and fun-seeking (sensitivity towards novel rewards) while the BIS is composed of one scale [39]. Previous research with adolescents has shown higher levels of substance use is associated with high BAS scores and low BIS scores [40].

Barratt Impulsiveness Scale (BIS-11) [41] was administered to the child and examines impulsiveness using three subscales: attentional, motor, and non-planning impulsiveness, with higher scores indicating a higher likelihood of impulsive behaviors. Higher BIS-11 scores are associated with earlier onset of substance use and a higher likelihood of abuse/dependency issues later on in life [42].

Measure of Eysenck’s Big 5 personality traits measures openness to experience, conscientiousness, extraversion, agreeableness, and neuroticism [43]. Big Five personality traits, in particular extraversion, neuroticism and openness, were important correlates of adolescent substance use [44,45].

### 2.3. Statistical Analysis 

Frequencies or means and standard deviations of demographic, social environmental, and psychological/functional characteristics of youth were tabulated by group, and the differences between FH+ and FH- groups tested using chi-square or *t*-tests. Fisher’s exact test was used for categorical measures with cell counts < 5. Statistics were calculated among observations with non-missing data. The proportion of observations with missing data was generally low. For scale measures, we used mean responses of observed items when at least 60% of total items had non-missing values. 

To address potential confounding of relationships between FH+ and psychological or functional characteristics by demographic factors, linear or logistic regression models were fit adjusting for subject sex, age and race/ethnicity. To address potential misclassification of FH+, these models were refit, excluding subjects who: (a) were classified as FH-, but for whom a proxy response indicated a potential SUD in a biological non-participant parent; or (b) were classified as FH+, but for whom the parent with SUD was other than the biological mother or father.

To empirically identify sub-groups of individuals with similar psychological and functional characteristics across the measured domains, latent profile analysis (LPA) [46] used the continuous psychological variables, standardized to improve interpretability. LPA is a variant of latent class analysis with continuous indicators of class membership. LPA models with 2–4 profiles were fit, and compared using fit indices (see Table S1). The association of FH+ or demographic characteristics with class membership were tested using multinomial logistic regression in a one-step approach [47,48]. Odds ratios (OR) of group membership were estimated for each predictor. Statistical analyses were conducted using SAS 9.4 (SAS Institute Inc., Cary, NC, USA) and Mplus 7.11.

## 3. Results

Overall, participants included 142 youth age 12–17 (mean = 15.1; SD = 1.3) during the current wave (wave 3), with 48% female. The demographic characteristics of FH+ and FH- participants are shown in Table 1. Participants were primarily Hispanic or Black, with low household income. FH+ and FH- participants did not differ significantly with respect to demographic characteristics.

### 3.1. Youth Family and Social Environment

The family and social environmental characteristics of participants are shown in Table 2. FH+ subjects lived in larger households than did FH- subjects (mean (SD) of 4.9 (1.9) vs. 4.3 (1.5) household members, *p* = 0.02). However, the numbers of household members under age 18 and the presence of the subject’s biological parents did not significantly differ, with the majority living with one biological parent. The parents of FH+ children reported higher levels of psychological aggression (*p* = 0.02), but not physical aggression, neglect, or non-violent discipline, and parental monitoring did not differ between the groups. FH+ subjects had been exposed to a higher number of ACEs, even after excluding parental SUD (mean (SD) = 4.6 (1.6) versus 4.0 (1.3); *p* = 0.01).

### 3.2. Youth Psychological Characteristics, Psychopathology, and Functioning

Adjusted coefficients or odds ratios (OR) for the association of FH+ with psychological characteristics and measures of function are shown in Table 3. FH+ subjects had lower reported school grades according to parental report (adjusted beta = 0.42; *p* = 0.004 on a 1–5 scale from best to worst); school grades on the past report card were also poorer among FH+ subjects according to child report (*p* = 0.05). FH+ subjects were more likely to have a history of probable diagnosis of externalizing disorder (OR (95% CI) = 3.41 (1.15, 10.1), and had marginally (0.05 < *p* < 0.1) higher levels of attentional impulsiveness on the BIS-11 and marginally lower levels of extroversion and agreeableness. FH+ and FH- youth did not differ in IQ, anxiety, motivation, or Vanderbilt Scale ADHD symptom measures. Excluding the 15 subjects with potentially misclassified FH+ gave similar results.

### 3.3. Psychological Characteristic Profiles

The continuous psychological variables included in the LPA are indicated in Table 3. Table S1 shows the fit indices for the two through four class models. The Akaike information criterion (AIC), Bayesian information criterion (BIC), and adjusted BIC suggested that models with more classes provided better fit than those with fewer latent classes, although the Lo-Mendell-Rubin adjusted LRT suggested that 2 classes were sufficient. We chose a 3-class solution to balance these considerations and because this produced reasonable sample size per class (>10% of the sample) and interpretable profiles. Entropy of the 3-class model was 0.9 indicating strong discrimination of the latent classes.

**Figure 1:**
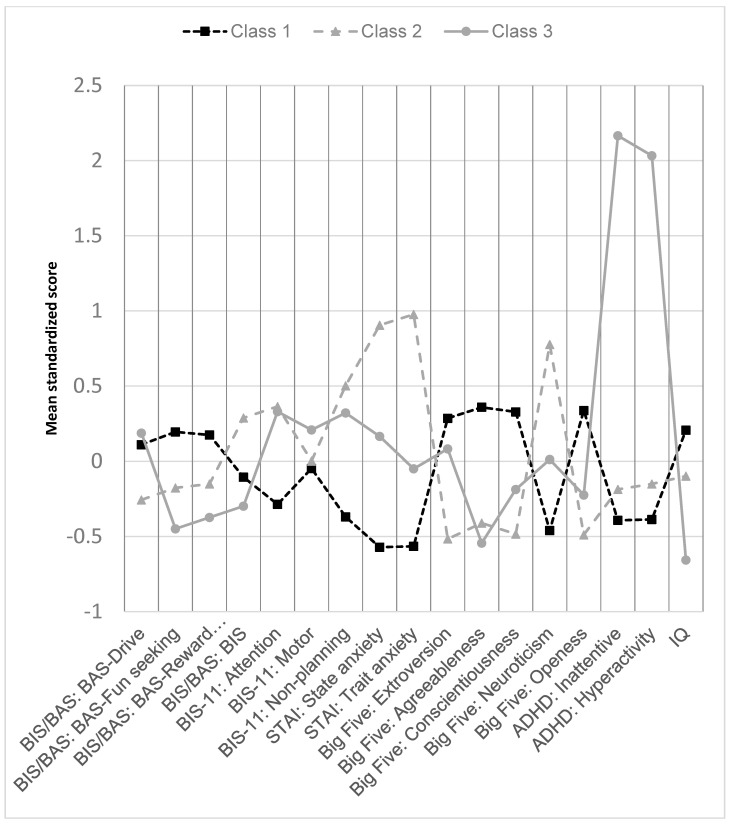
Latent profile analysis—3 class model—mean scores for indicators in each class.

The profiles describing each of the three classes are displayed in Figure 1. An “Anxious” class comprising 32% of the population was characterized by high state and trait anxiety as well as neuroticism. An “Inattentive” class comprising 13% of the population was characterized by high levels of both inattentive and hyperactive symptoms of ADHD and by lower IQ. The remaining 55% was characterized by relatively moderate mean levels of all characteristics and is labeled as “Typical”. In multivariable multinomial logistic regression analyses (Table 4), age, race/ethnicity, gender, and FH+ were not associated with being in either the “Anxious” or “Inattentive” class relative to the “Typical” class. Female participants did, however, have more than four times the odds of belonging to the “Anxious” versus the “Inattentive” class (OR = 4.48; 95% CI = 1.04–19.3; *p* = 0.04). Neither FH+, race/ethnicity, or age were associated with significant differences in the odds of being “Anxious” versus “Inattentive” (all *p*-values > 0.2).

## 4. Discussion

We compared the family/social and psychological characteristics of demographically similar FH+ and FH- youth from a longitudinal study of parental CJSI in a primarily low-income, minority urban population. Several key findings emerge. While the groups were similar overall in family and social and economic environments, the FH+ group had evidence of greater family environment adversity, including a higher parental psychological aggression score and higher number of ACEs, including those other than parental SUD. 

Additionally, the FH+ youth had lower grades and a higher prevalence of a history of externalizing disorders, but did not differ significantly from FH- youth on most other psychological measures. Considering the youth psychological measures collectively, the observations grouped into three latent classes: (a) high anxiety and neuroticism, (b) inattention and hyperactivity; and (c) typical levels of all measures. Gender, but not FH+ was associated with belonging to the ‘anxious’ versus ‘inattentive’ class. 

The lack of differences in psychological measures between groups in our study contrasts with the findings of a prior study focused on minority youth that observed differences in behavioral traits associated with increased risk of SUD in FH+ versus FH- youth [19]. This may be due to methodological differences, specifically, the prior study excluded potential participants from the FH- but not the FH+ group based on the presence of externalizing or anxiety disorders and ADHD [19], whereas we applied identical criteria for selecting youth from each group. Additionally, unlike our study, they found lower parental socioeconomic status in the FH+ group [19], suggesting that familial environmental factors may also have contributed to group differences, as opposed to FH+ alone. On the other hand, exposure to childhood adversity was higher in FH+ in our study, and has previously been associated with increased risk of developing SUD across the lifespan [49]. Our findings are consistent with those of another recent study reporting that family history of psychopathology (including SUD) was not associated with differences in delay discounting behavior at ages 9–11, but rather sociodemographic differences primarily accounted for observed behavioral differences [50].

The few differences we found between the FH groups, namely higher prevalence of ACEs, lower grades, and higher prevalence of a history of externalizing disorders within FH+, are consistent with known SUD risk factors. Previous studies have demonstrated that: ACEs were associated with earlier age of initiating opioid use [51], that ACEs with mood disorders contribute additively to the risk of SUD [52], and that children exposed to severe or chronic stress [10,11] or with poor family environment and relationship [3,12,13] have increased risk of SUD. In addition, familial transmission of individual behavioral traits, such as impulsivity, risk-seeking and emotional dysregulation, which are seen in individuals with lower grades and higher prevalence externalizing disorders, have also been proposed to contribute to SUD risk [15,16,17]. 

Our LPA analysis, that segregated participants based on their psychological characteristics, captures these emotion dysregulation and attention dimensions. However, the LPA-based profiles did not significantly correspond to our FH+/FH- classification. Thus, the results suggest that, at this point during their development, FH-based risk for SUD is more closely related to parent-child relationships, observed in this study as parental aggression score and higher ACEs, than to youth psychological or behavioral characteristics. If further validated, this would support prevention strategies involving family-relation interventions. Our previous finding suggesting that FH+ is associated with changes in stress reactivity among a subset of adolescents from this cohort completing a task-based assessment [53] further underscores the potential importance of strategies to mitigate stressful life events and circumstances.

The strengths of this study include a population drawn from a well-characterized cohort with both youth and parent informants. Both the FH+ and FH- participants were largely minority and had low SES, with a high prevalence of parental CJSI, representing an understudied population at high overall risk for SUD. The classification of FH+ was based on 3 waves of longitudinal data, decreasing misclassification, and longitudinal data on the history of exposure to ACEs and youth history of psychopathology was also available. We used LPA, in addition to comparisons of individual characteristics, to determine whether FH+ was related to any specific profile defined by a range of psychological and functional characteristics. While this did not distinguish FH+/FH- at this point in the youths’ development, this wealth of information may help determine more precisely which of these factors will contribute to the familial transmission of SUD as these youth progress through the adolescent period of high-risk behavior. 

Limitations of the data should be acknowledged. Given the sample size of 73 FH+ and 69 FH- youth, we may have lacked power to observe true differences between groups. It is also possible that FH groups differed on characteristics other than those included, for example peer substance use. Since this sample is unique in that it targeted those with parental CJSI, it can serve as a starting point for further larger investigations but may be of somewhat limited generalizability. However, given that our sample was drawn specifically to be similar with regard to social and environmental factors, our finding of fewer differences between the FH groups relative to previous studies may also be due to the diminished impact of such potential confounding factors. Likewise, while the instruments examined were selected for their relevance to adolescents and to adolescent substance use, psychometric properties in this highly selected population may differ from those previously reported in more general samples. Additionally, it is possible that some youth classified as FH- had familial risk for SUD through either their non-participating biological parent or other close relatives. However, sensitivity analyses excluding subjects in whom proxy information suggested potential SUD in a non-participating biological parent did not alter these findings. Finally, the parental SUD in our sample was heterogenous as they used different types of substances. However, an SUD involving any class of substances confers increased risks among relatives for all other classes of SUD [2], indicating a common liability that is shared across the disorders.

In conclusion, only subtle differences in psychological characteristics were found between demographically similar FH+ and FH- youths from a minority population. Some differences in childhood environment and interpersonal factors indicative of parenting style differed across the groups. If this result is further validated, it would have important implications for prioritizing family-relation interventions as well as those that counter negative effects of childhood adversity associated with socioeconomic disadvantage as prevention strategies for SUD in FH+ youth. Future research should carefully address the potential for socioeconomic or demographic confounding in comparisons of FH+ versus FH- youth, while incorporating increased sample sizes and diverse populations from a variety of contexts.

## Figures and Tables

**Table 1:** Demographic characteristics of FH+ and FH- youth.

Characteristic	FH- (*N* = 69)	FH+ (*N* = 73)	*p*-Value ^a^
	Mean (SD)	Mean (SD)	
Age	15.1 (1.33)	15.19 (1.34)	0.65
	N(%)	N(%)	
Female	34 (49.28)	34 (46.58)	0.75
Race/Ethnicity			0.62
Hispanic	42 (60.87)	41 (56.2)	
Black	15 (21.74)	21 (28.77)	
Other/multiple races/unknown	12 (17.39)	11 (15.07)	
Parental Education ^b^			0.17
Did not complete HS	19 (27.94)	23 (32.86)	
HS graduate/GED	17 (25.00)	16 (22.86)	
Some college	22 (32.35)	13 (18.57)	
Associate degree or higher	10 (14.71)	18 (25.71)	
Parent is currently employed ^b^	39 (57.35)	34 (48.57)	0.30
Annual household income ^b^			0.96
≤$15,000	24 (35.82)	24 (34.29)	
$15,000–50,000	30 (44.78)	33 (47.14)	
≥$50,000	13 (19.40)	13 (18.57)	

^a^ *p*-value from chi-square or *t*-test; ^b^ Percentages are based on observations with non-missing information. Information was missing for parental education (n = 4); parental employment (n = 4); and annual household income (n = 5).

**Table 2:** Family and social environmental characteristics of FH+ and FH- youth.

Characteristic	FH- (*N* = 69)	FH+ (*N* = 73)	*p*-Value ^a^
	N(%) or mean (SD)	N(%) or mean (SD)	
Household composition			
Total number of people	4.30 (1.45)	4.94 (1.86)	**0.02**
Number of people under age 18	2.15 (1.18)	2.41 (1.39)	0.22
Number of biological parents			0.38
0	1 (1.45)	4 (5.48)	
1	49 (71.01)	47 (64.38)	
2	19 (27.54)	22 (30.14)	
Parent-child relationship			
Parental monitoring	17.41 (2.30)	17.38 (2.23)	0.95
Parent Child Conflict Tactics Scale, Non-Violent Discipline ^b^	5.21 (3.24)	6.00 (3.49)	0.17
Parent Child Conflict Tactics Scale, Psychological aggression ^b^	4.08 (3.03)	5.33 (3.42)	**0.02**
Parent Child Conflict Tactics Scale, Physical aggression	0.66 (1.62)	0.60 (1.62)	0.84
Parent Child Conflict Tactics Scale, Neglect ^b^	0.35 (1.12)	0.60 (1.27)	0.23
Perceived availability of substances as fairly or very easy to get			
Cigarettes ^b^	37 (56.92)	39 (56.52)	0.96
Alcohol ^b^	35 (54.69)	35 (50.72)	0.65
Marijuana ^b^	32 (50.00)	36 (54.55)	0.60
Crack or cocaine ^b^	17 (29.31)	10 (15.63)	0.07
Youth perceived harm of substances			
Smoking one or more pack cigarettes/day ^b^	50 (73.53)	48 (65.75)	0.32
Using marijuana 1–2 times/week ^b^	21 (30.88)	12 (16.90)	0.053
Having 5+ alcoholic drinks 1–2 times/week ^b^	34 (50.00)	40 (54.79)	0.57
Trying heroin 1–2 times ^b^	41 (62.12)	32 (46.38)	0.07
Peer Relations (higher = better peer)	5.70 (1.90)	5.53 (1.80)	0.60
Number of ACEs ^c^	4.01(1.31)	5.45 (1.64)	**<0.001**
Number of ACEs other than parental SUD ^c^	4.01 (1.31)	4.63 (1.61)	**0.01**

^a^ *p*-value from chi-square or *t*-test; ^b^ Percentages are based on observations with non-missing information. Information was missing for Parent Child Conflict Tactics Scale: Non-violent discipline (n = 1), Psychological aggression (n = 2), and Neglect (n = 1); perceived availability of substances: cigarettes (n = 8), alcohol (n = 9), marijuana (n = 12), and crack or cocaine (n = 20); perceived harm of substances: cigarettes (n = 1), marijuana (n = 3), alcohol (n = 1), and heroin (n = 7). ^c^ Based on information collected during parent study (S&J). Bold values indicate *p* < 0.05.

**Table 3:** Measures of functioning and psychological characteristics in FH+ and FH- youth.

Characteristic	FH- (*N* = 69)	FH+ (*N* = 73)			
Functioning	Mean (SD)	Mean (SD)	Beta ^a^	95% CI	*p*-value
Current/Just completed grade ^b^	8.90 (1.54)	8.87 (1.40)	−0.13	(−0.39, 0.14)	0.34
Grades on last report card, child report (1–7, best to worst) ^b^	3.00 (1.46)	3.46 (1.44)	0.46	(0.002, 0.93)	**0.05**
Child’s grades past year of school, parent report (1–5, best to worst) ^b^	2.06 (0.94)	2.51 (0.89)	0.42	(0.13, 0.72)	**0.004**
	N (%)	N (%)	OR ^a^	95%CI	
Child history of psychopathology ^d^					
Internalizing disorder(s)	34 (49.28)	35 (47.95)	0.95	(0.49, 1.83)	0.87
Externalizing disorder(s)	5 (7.25)	15 (20.55)	3.41	(1.15, 10.1)	**0.03**
Child history substance use					
Any history of substance use	10 (14.49)	16 (21.92)	1.73	(0.66, 4.50)	0.26
Regular substance use	1 (1.45)	3 (4.11)	0.36	(0.04, 3.70)	0.39
	Mean (SD)	Mean (SD)	Beta ^a^	95% CI	
KBIT:IQ composite standard score ^b–d^	91.59 (14.14)	93.44 (11.84)	1.46	(−2.81, 5.74)	0.50
State and trait Anxiety					
STAI State score ^c^	32.74 (8.58)	34.61 (8.69)	1.83	(−0.94, 4.61)	0.20
STAI Trait score ^c^	35.49 (8.96)	36.70 (9.07)	1.28	(−1.61, 4.16)	0.39
Vanderbilt: ADHD symptoms					
Number of Hyperactivity symptoms ^b,c^	1.23 (2.26)	1.43 (2.01)	0.17	(−0.53, 0.87)	0.63
Number of Inattentive symptoms ^b,c^	1.09 (2.37)	1.75 (2.38)	0.57	(−0.19, 1.34)	0.14
BIS/BAS					
BIS/BAS: BAS Drive score ^c^	10.35 (2.10)	10.48 (2.19)	0.13	(−0.57, 0.82)	0.72
BIS/BAS: BAS Fun seeking score ^b,c^	11.37 (1.48)	11.22 (1.75)	−0.12	(−0.65, 0.40)	0.64
BIS/BAS: BAS reward responsiveness score ^c^	15.73 (1.94)	15.66 (2.02)	−0.10	(−0.74, 0.54)	0.77
BIS/BAS: BIS total score ^c^	18.24 (2.41)	18.14 (2.89)	−0.11	(−0.97, 0.74)	0.80
BIS-11					
BIS-11 Attentional impulsiveness ^c^	15.68 (2.87)	16.53 (3.15)	0.92	(−0.06, 1.91)	0.07
BIS-11 Motor impulsiveness ^b,c^	21.33 (3.83)	21.83 (4.47)	0.47	(−0.88, 1.82)	0.50
BIS-11 Non-planning impulsiveness ^c^	27.00 (4.55)	27.88 (5.00)	0.98	(−0.56, 2.52)	0.21
Big 5 Personality					
Big 5 Extroversion ^c^	26.86 (4.10)	25.56 (3.92)	−1.28	(−2.57, 0.02)	0.05
Big 5 Agreeableness ^c^	33.10 (5.11)	31.76 (3.88)	−1.38	(−2.84, 0.09)	0.07
Big 5 Conscientiousness ^c^	29.18 (5.09)	28.49 (4.39)	−0.79	(−2.33, 0.75)	0.32
Big 5 Neuroticism ^c^	21.46 (4.78)	22.48 (4.35)	1.11	(−0.31, 2.54)	0.13
Big 5 Openness ^c^	34.21 (5.17)	33.56 (4.75)	−0.69	(−2.30, 0.92)	0.40

^a^ Betas and ORs are adjusted for subject sex, race/ethnicity, and age. ^b^ Based on observations with non-missing information. Information was missing for: current grade (n = 1); child and parent report of grades on last report card (n = 3); IQ composite score (n = 8); ADHD hyperactivity (n = 2) and inattentive (n = 1) symptoms; BAS fun seeking score (n = 1); and BIS-11 motor impulsiveness (n = 1). ^c^ Variables included in latent profile analysis. ^d^ Based on information collected during parent study (S&J). Bold values indicate *p* < 0.05.

**Table 4:** Multinomial LR ORs for class membership associated with participant characteristics.

	“Anxious” vs. “Typical”				Inattentive vs. “Typical”			
Characteristic	OR	95% CI		*p*-Value	OR	95% CI		*p*-Value
Age	1.23	0.90	1.68	0.21	1.01	0.65	1.54	0.98
Hispanic	1.00	(Ref)		--	1.00	(Ref)		--
Black	0.79	0.26	2.41	0.68	1.70	0.50	5.80	0.40
Other	0.55	0.16	1.91	0.35	1.00	0.22	4.60	1.00
Female ^a^	1.53	0.60	2.45	0.92	0.34	0.09	1.26	0.11
FH+	1.04	0.45	2.45	0.92	1.37	0.44	4.28	0.58

^a^ Differed significantly for “Anxious” vs. “Inattentive” (OR = 4.48; 95% CI = 1.04–19.3; *p* = 0.04); *p* > 0.2 for all other characteristic comparisons between “Anxious” and “Inattentive”.

## Data Availability

After the acceptance for publication of the main findings from the final data set de-identified study data will be shared upon request with qualified researchers, subject to the terms of a data sharing agreement.

## Supplementary Materials

The following supporting information can be downloaded at: https://www.mdpi.com/article/10.3390/children11060671/s1, Table S1: Fit indices for 2–4 class latent profile models.
