# Supplementary material for: Characteristics of Adolescents with and without a Family History of Substance Use Disorder from a Minority Cohort
Source: Children (Basel). 2024 May 31;11(6):671. doi: 10.3390/children11060671 (PMC11201943; doi:10.3390/children11060671)
Supplement: Supplementary file 1 [file children-11-00671-s001.zip › children-2979664-supplementary.pdf]

Table S1. Fit indices for 2-4 class latent profile models.

	2 classes	3 classes	4 classes
AIC	6642.0	6484.1	6416.6
BIC	6796.1	6691.5	6677.3
Sample-Size adjusted BIC	6631.6	6470.0	6398.8
Lo-Mendell-Rubin adjusted LRT	0.002	0.23	0.17
Proportion in smallest class	35.4%	12.6%	7.6%
Entropy	0.87	0.90	0.93

AIC = Akaike information criterion; BIC = Bayesian information criterion; LRT= likelihood ratio test.
